# Short heatwaves during fluctuating incubation regimes produce females under temperature-dependent sex determination with implications for sex ratios in nature

**DOI:** 10.1038/s41598-017-17708-0

**Published:** 2018-01-08

**Authors:** A. W. Carter, B. M. Sadd, T. D. Tuberville, R. T. Paitz, R. M. Bowden

**Affiliations:** 1 School of Biological Sciences, Illinois State University, Normal, IL United States; 2 University of Georgia’s Savanah River Ecology Lab, Aiken, SC United States

## Abstract

Patterns of temperature fluctuations in nature affect numerous biological processes, yet, empirical studies often utilize constant temperature treatments. This can limit our understanding of how thermally sensitive species respond to ecologically relevant temperatures. Research on turtles with temperature-dependent sex determination (TSD) provides good examples of this, since nest temperatures from many populations rarely exceed those necessary to produce females under constant laboratory conditions. We hypothesized that exposure to brief periods of warm temperatures (i.e., heat waves) are integral to sex determination in species with TSD, which requires tests that move beyond constant temperatures. We exposed *Trachemys scripta* embryos from multiple populations and across the nesting season to heat waves of variable durations and quantified sex ratios. We found that embryos from all populations were highly sensitive to brief exposures to female producing temperatures; only 7.9 days of exposure produced a 50:50 sex ratio, but the response varied across the nesting season. From these findings, a model was developed to estimate sex ratios from field temperature traces, and this model outperformed traditional methods. Overall, these results enhance our understanding of TSD and emphasize the importance of using biologically relevant temperatures when studying thermally sensitive processes.

## Introduction

Temperature affects a wide range of biological processes, from the biochemical action of enzymes to cueing the onset of mass migrations^1–3^. As a result, considerable effort has been directed towards understanding and predicting the effects of temperature on these functions. However, the thermal conditions used in empirical and theoretical studies are often oversimplified (e.g. constant temperature treatments and low temporal resolution aggregate statistics, respectively), despite recent evidence demonstrating that patterns of temperature fluctuations can significantly alter predicted or actual biological responses^4–6^. Given this, investigations of thermally sensitive processes should be conducted using more realistic conditions. This is particularly necessary in light of ongoing climate change, which, in addition to increases in average temperatures, is manifesting as increased thermal variability and extreme weather events like heat waves^7–9^. Understanding how organisms respond to dynamic thermal conditions that more closely approximate those experienced in nature is vital to advancing the field of thermal biology and better estimating species responses to climate change.

Ectotherms are very likely to be impacted by climate change^10^, and research that enhances predictions of how these species respond to thermally fluctuating environments is particularly timely. Species with temperature-dependent sex determination (TSD), where offspring sex is determined by incubation temperature, may be particularly susceptible due to the risk of completely sex biased populations^11^. Turtles have emerged as model TSD organisms, and in these species, embryos experience incubation temperatures that fluctuate concurrent to ambient temperatures^12^. Yet the foundation of what is known about TSD is principally based on constant temperature incubation studies conducted in the lab^13^. These studies have determined that, in the most common form of TSD, females are produced at warm temperatures and males are produced at cool temperatures^14^. There is a pivotal temperature (Tpiv) where a 50:50 population-wide sex ratio is produced, and a range of temperatures over which mixed sex ratios are produced (the transitional range of temperatures). These constant temperature incubation studies have also found that sex is labile during approximately the middle third of development, which is termed the thermal sensitive period (TSP)^15–19^. Mechanistically, it is thought that warm temperatures during the TSP increase oestradiol concentrations in the bipotential gonad by increasing aromatase expression and this subsequently triggers ovarian differentiation^20–22^. These laboratory studies have also provided a relatively detailed understanding of how constant incubation temperatures influence sex ratios in turtles. Unfortunately, sex ratio patterns derived from laboratory studies do not reflect sex ratio patterns in the field, hindering accurate predictions of sex ratios in nature.

Studies that focus on how natural incubation temperatures affect sex ratios often report that more females are produced than would be expected under laboratory conditions. For example, in *Chrysemys picta*, incubation temperatures of natural nests averaged 25.5 °C during the TSP and yielded mostly female hatchlings, though incubation at that temperature in the lab would produce only males (*C. picta* Tpiv ~28.5 °C)^23^. In the same population, longitudinal sex ratio estimates average ~36% female despite average air temperatures ranging from ~20–26.5 °C, minimally 2 °C lower than the Tpiv for *C. picta*, over the last 23 years^24,25^. In these examples, annual hatchling cohort sex ratios are compared to temperatures averaged over the entire developmental period or within the middle third of development estimated by direct calculations or using July temperatures as a proxy^23–25^. Indeed, aggregate temperature statistics have traditionally been used to estimate hatchling sex ratios, yet they often underestimate the production of females compared to laboratory incubations^26–28^. Mathematical models have been developed to address the tendency to observe more females under fluctuating incubation conditions than constant temperatures of the same mean^29,30^. The Constant Temperature Equivalent (CTE) model^29,30^ converts fluctuating conditions to a single constant temperature equivalent that can be compared to the Tpiv for any particular species to estimate sex ratios. Because development is faster at warmer (female) incubation temperatures, during a given unit of time, a greater proportion of development is achieved at female producing temperatures than at male producing temperatures. The CTE accounts for temperature-dependent developmental rates of embryos and subsequently estimates the temperature at which half of development occurs above and half below. The CTE model does an excellent job at predicting sex ratios under consistent laboratory fluctuations^29–33^; however, the CTE does not perform as well when predicting sex ratios from the field as the CTEs estimated from most nests still fall below the Tpiv^23–25^. The disparity between patterns of sex determination in the laboratory versus the field underscores the necessity of better characterizing how more realistic thermal conditions during incubation affect sex determination.

**Figure 1:**
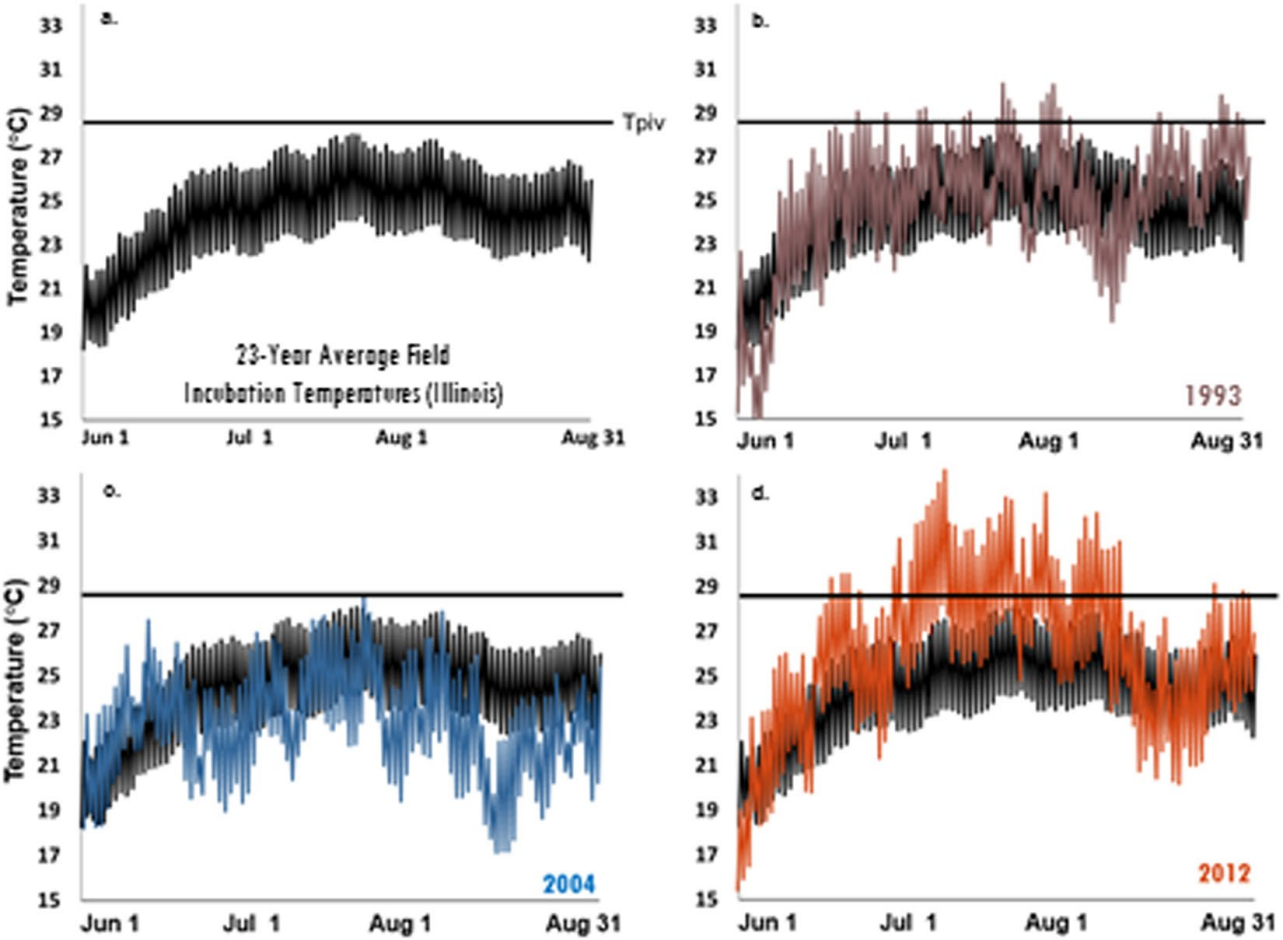
Sub-surface soil temperatures from Peoria, Illinois^34^. In all figures, the Tpiv of *Trachemys scripta* is demarcated with a flat line. (**a**) The 23-year average soil temperatures during the breeding and incubation season never rise above the Tpiv. Temperature traces from individual years are compared to the 23-year average in panels B–D. Panel B is a representative thermal trace of temperatures most frequently observed. Note that temperatures periodically rise above the Tpiv for brief periods of time. Panel C is a representative cool year, and panel D. is an anomalous warm year.

At our Illinois field site, the 23-year average of maximum sub-surface soil temperatures^34^, which approximate nest temperatures, is lower than the Tpiv of our focal turtle species with TSD, *Trachemys scripta* (*T. scripta* Tpiv ~28.5 °C^35^; Fig. 1a). Sex ratio estimates based on this long-term average would indicate the production of 100% males, rendering the production of females at the field site enigmatic. Mark-recapture efforts over the last 12 years show that females are recruited into the breeding population regularly, thus conditions at the site must be conducive to female production in at least some years. While the long-term average of soil temperatures is below the Tpiv, soil temperatures periodically and briefly rise above the Tpiv (average 8 days per year;^34^, Fig. 1b). Because of the historic use of constant incubation temperatures or conditions utilizing consistent diurnal fluctuations for the entirety of development^13^, we have not been able to discern how much exposure to female producing conditions during the TSP is actually necessary to trigger ovary development. If only a relatively short amount of time is needed at female producing temperatures to trigger ovary development, then females could be produced at conditions that otherwise have averages (or CTEs) well below the Tpiv. We hypothesize that these “heat waves” (i.e. brief periods where temperatures exceed the Tpiv) are integral to the determination of sex in species with TSD. We predict that turtle embryos are highly sensitive to brief durations of exposure to temperatures above the Tpiv, and that ovary determination can be triggered after only a few days of exposure to warm temperatures. Such a finding would substantially alter our understanding of how sex is determined in species with TSD, and would serve as a case study for emphasizing the importance of thermal realism.

**Figure 2:**
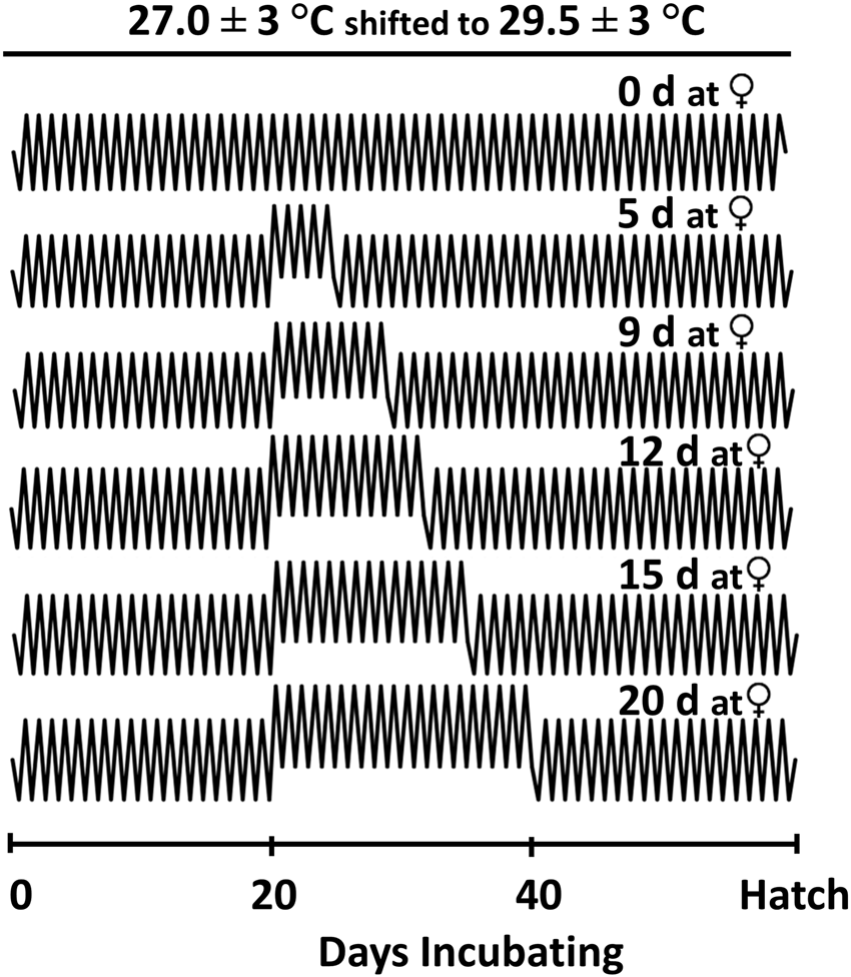
A selection of representative incubation treatments. In all treatments *Trachemys scripta* embryos were incubated at male producing temperatures (27 ± 3 °C) for the first 20 days of incubation, then eggs were moved to female producing temperatures (29.5 ± 3 °C) for variable durations, after which they were returned to the male temperatures for the remainder of incubation. See the methods for all the incubation duration treatments utilized in each population.

This research integrates (I) empirical and (II) modeling approaches to demonstrate that the inclusion of naturalistic thermal conditions can greatly improve our understanding of thermal physiology and enhance related thermal models. (Ia) We characterized the amount of exposure to female producing temperatures necessary to trigger ovary determination in *T. scripta* through controlled laboratory incubations that varied exposure to warm (female producing) incubation temperatures (called ‘heat waves’ throughout for simplicity) (Fig. 2, Table 1). (Ib) We determined how multiple populations of *T. scripta* from different climates may vary in their response to heat wave exposure. Because turtle embryos from across the species’ distribution experience different climates on average, yet possess a similar Tpiv^25,26,36–39^, it is possible that embryos from northern and southern populations respond differently to heat waves. (Ic) We also determined if embryos from across the nesting season respond differently to heat waves during incubation. There is a seasonal increase in the concentration of maternally derived oestrogens in *T. scripta* yolks, which coincides with an increase in the production of females^35^. Because female differentiation is thought to be triggered by sufficient accumulation of oestradiol in the gonads^19,21,22^, embryos from late season eggs with high maternal oestrogen concentrations may require less exposure to female producing temperatures to trigger ovary development than early season eggs.

**Table 1:** Incubation treatments.

Exposure Duration (Days)	Average Incubation Temperature (°C)	Average CTE (°C)
0	27.0	27.8
3	27.1	27.9
5	27.2	28.0
6	27.2	28.0
7	27.3	28.1
9	27.3	28.1
10	27.4	28.2
12	27.5	28.2
15	27.6	28.4
20	27.8	28.5
25	27.9	28.7
30	28.1	28.9

Using the results from the incubation studies, we then developed a model to better predict sex ratios from field thermal traces. This model estimates sex ratios by counting the number of days within the TSP that embryos experience female-producing temperatures. We sought to (IIa) determine the performance of the model relative to the CTE approach using a long-term dataset of sex ratios measured in the field with corresponding soil temperature traces, (IIb) characterize historic sex ratios at each of our three populations, and (IIc) describe the relative impact of the seasonal shift in sex ratios mediated by maternal oestrogen deposition under concurrent seasonal shifts in field temperatures across time.

## Results

### Empirical Study

#### Oestradiol Concentrations

Populations did not differ in concentrations of yolk oestradiol (average ± 1 S. E.; Illinois: 0.3 ± 0.25 ng/mL, South Carolina: 0.7 ± 0.26 ng/mL, Louisiana: 0.4 ± 0.25 ng/mL; F_2,51_ = 0.06, p = 0.95), confirming consistent collection of early season clutches. Early and late season Illinois clutches significantly differed in yolk oestradiol concentration, confirming seasonal collection of clutches (early season: 0.3 ± 0.25 ng/mL, late season: 4.6 ± 0.55 ng/mL; F_1,35_ = 66.15, p < 0.0001).

#### Population Sex Ratio Reaction Norm

**Figure 3:**
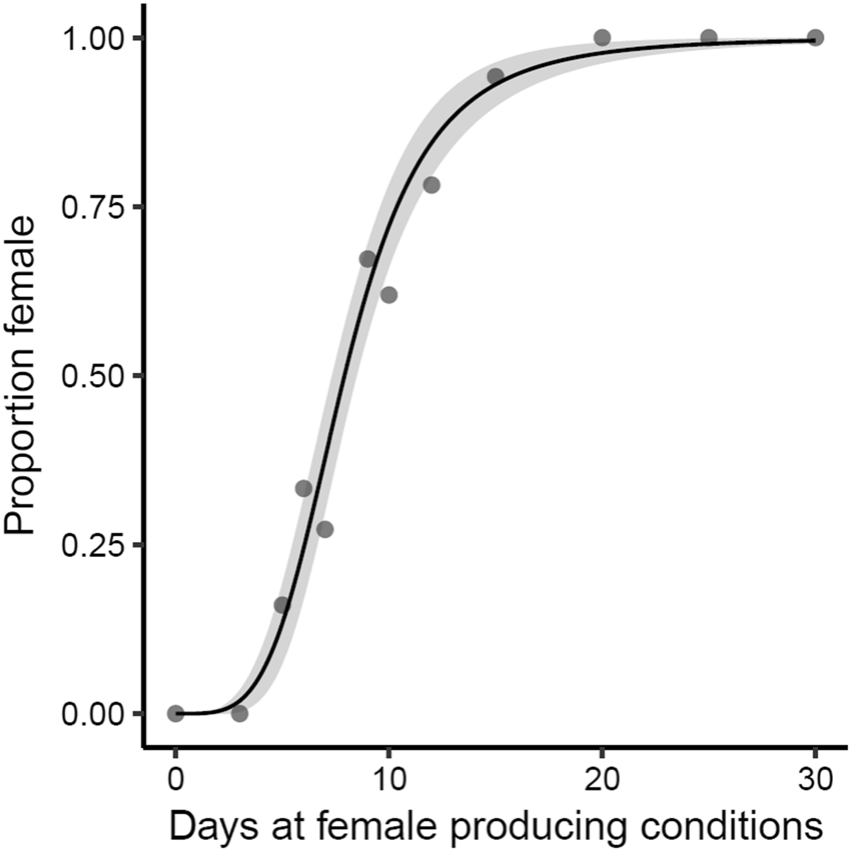
Populations did not differ in their responses to the incubation duration treatments. The curve depicts the population average sex ratio reaction norm. Points are proportions averaged across populations for each treatment and the ribbon is a 95% confidence interval.

For all populations, the control group (0 d) produced no females. Starting after only five days of exposure to female producing conditions, females were produced (mean = 0.16 female). The proportion of females increased with increased exposure until 20 days of exposure; treatments of 20, 25, and 30 days produced all females (Fig. 3). The proportion of females produced in each population by treatment are as follows (treatment: proportion female), Louisiana 0d:0, 5d:0.24, 9d:0.77, 12d:0.9, 15d:0.95, 20d-30d:1; South Carolina 0d:0, 5d:0.21, 9d:0.57, 12d:0.71, 15d:0.92, 20d-30d:1; Illinois 0-3d:0, 5d:0.05, 6d:0.33, 7d:0.27, 9d:0.64, 10d:0.62 12d:0.71, 15d:0.95, 20d:1. Allowing populations to individually vary in individual parameters did not significantly improve the model (Likelihood Ratio Test: χ^2^_4_ = 7.2, p = 0.13). Parameter estimates are provided in Table 2.

**Table 2:** Parameter estimates for the population and season models.

Model	Parameter	Estimate	Standard Error	95% Confidence Interval	
				Lower Limit	Upper Limit
**Population**	*a*	−4.06	0.35	−4.83	−3.30
	*b*	7.92	0.31	7.33	8.50
**Season**					
*Early*	*a*	−4.19	0.60	−5.37	−3.02
	*b*	8.43	0.40	7.64	9.21
*Late*	*a*	−4.48	0.77	−6.00	−2.97
	*b*	6.48	0.39	5.71	7.25

Note: ***a*** denotes the Hill Coefficient, the steepness of the reaction norm.

***b*** denotes the effective dose, the duration of exposure (days) to female producing conditions required to produce 50% females.

#### Seasonal Illinois Sex Ratio Reaction Norms

**Figure 4:**
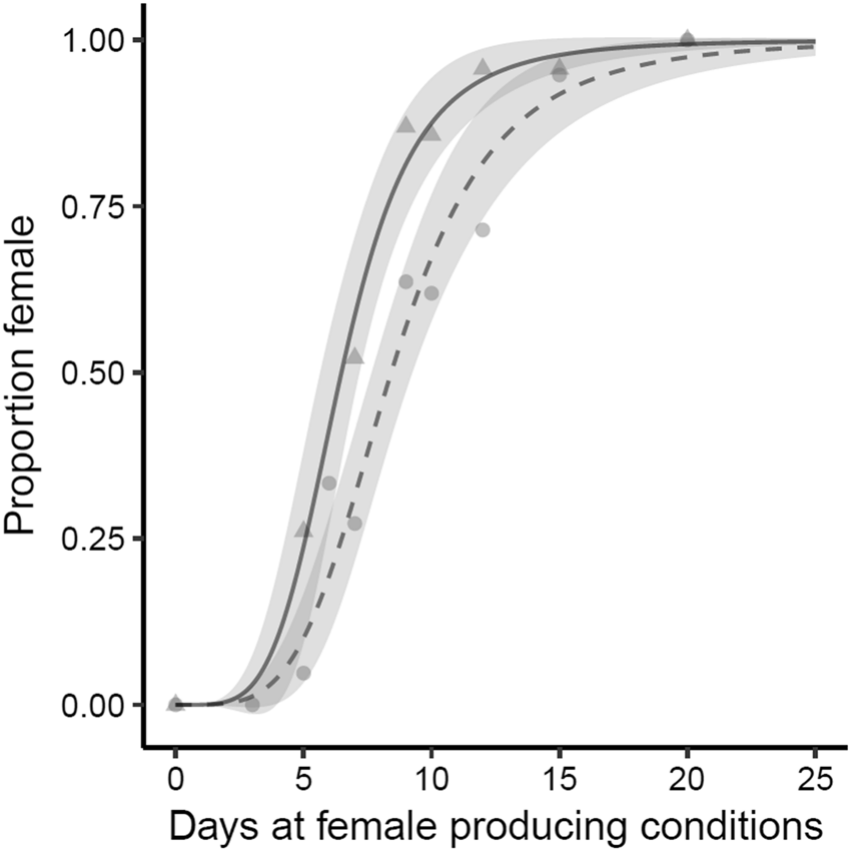
The proportion of females produced after a given incubation duration treatment significantly differed between early and late season individuals in Illinois. Late season clutches required less exposure to female temperatures to achieve a balanced sex ratio than early season clutches. The season specific sex ratio reaction norms are depicted by the curves (early season: dashed line, late season: solid line). Circles are early season proportions by treatment, and triangles are late season proportions by treatment. The ribbons correspond to 95% confidence intervals.

Within the Illinois population, inclusion of independent parameter estimates for season provided a better fit than the model that only specified incubation duration treatment (Fig. 4), demonstrating that embryos from early and late season clutches possess different sex ratio reaction norms (Likelihood Ratio Test: χ^2^_2_ = 14.58, p = 0.0007), presumably as a result of the seasonal difference in maternal oestrogen deposition^35^. These parameter estimates (Table 2) show that the differences in reaction norms between seasons are not due to differing Hill coefficients (*a*) but due to differences in *b*, the duration (days) of exposure to female producing conditions that produces 50% females.

### Modeling Study

#### Determining the Predictive Ability of the DDC Model

**Figure 5:**
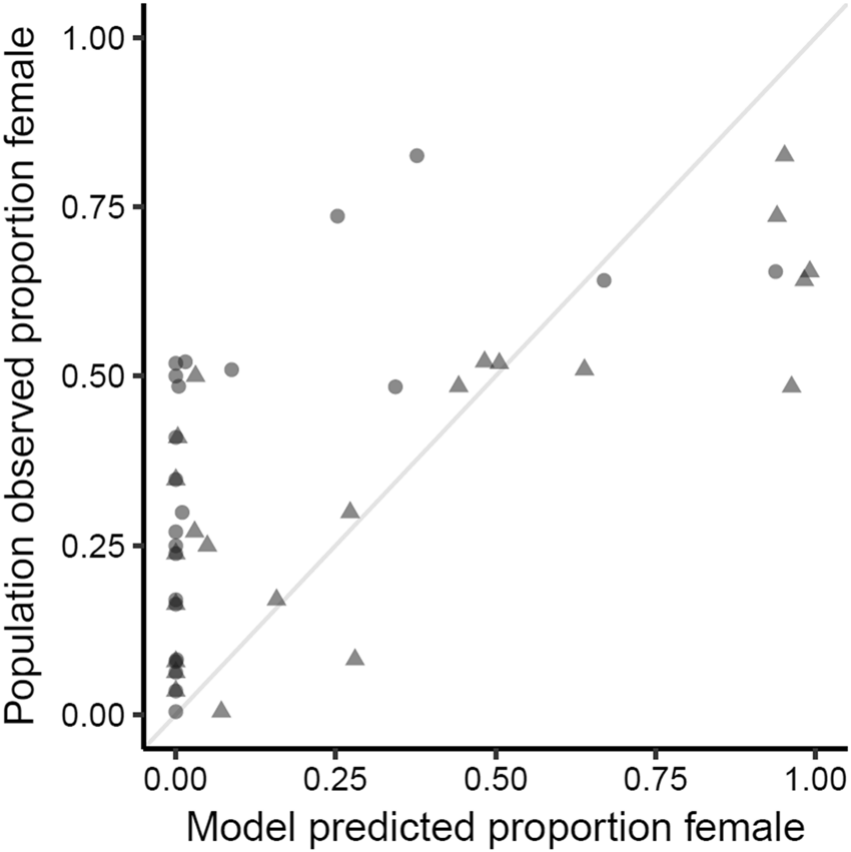
Predicted versus observed sex ratios in *C. picta*. Observed sex ratios are from a northern Illinois population, and each point denotes an average annual hatchling cohort sex ratio. Sex ratios predicted from the CTE model are denoted with circles, and those from the DDC Model are denoted with triangles. A 1:1 comparison line is depicted in grey. The DDC Model performs significantly better than the CTE model (see results).

The Daily Duration with CTE (DDC) Model fit the observed sex ratios significantly better than the CTE model (DDC Model R^2^ = 0.64, CTE model R^2^ = 0.35; Encompassing Test: F _1,20_ = 16.27, p = 0.0006; Fig. 5). Results from the Cox Test and the J Test corroborated these findings. Predictions based on the average were not plotted or analysed further as in all years this model predicted that no females would be produced. Since the DDC Model was developed in *T. scripta*, and the observed data are in *C. picta*, we ran these tests with both species’ specific *T0* (*T. scripta* = 16 °C, *C. picta* = 14 °C^31^ and found overall conclusions to be consistent regardless of the *T0* specified.

#### Estimating Sex Ratios

**Figure 6:**
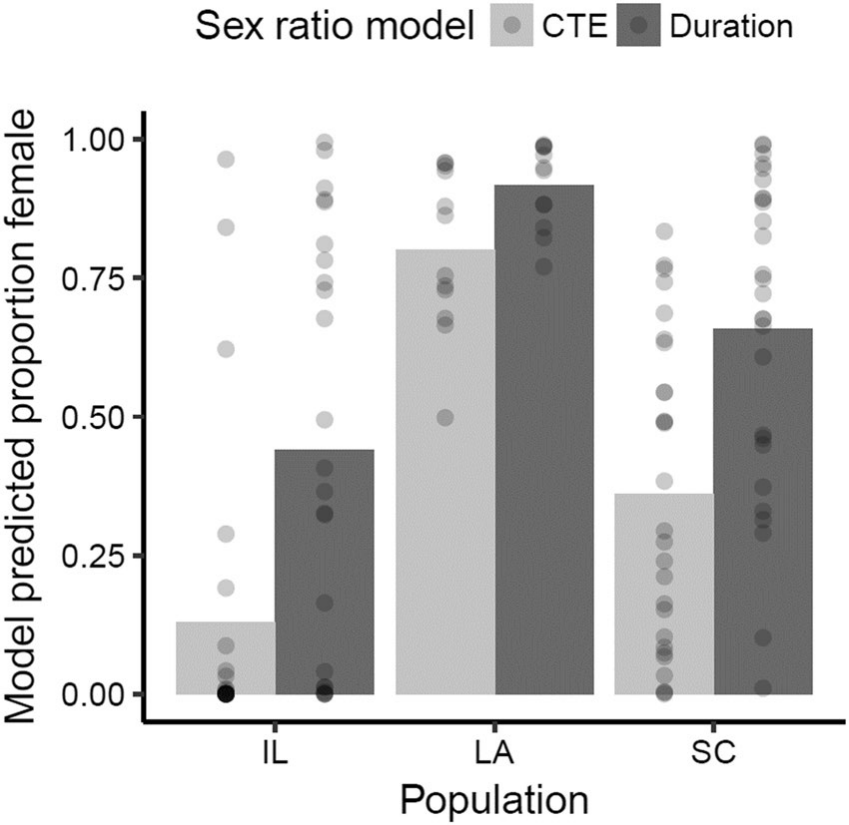
Inter-annual averages (bars) of hatchling cohort sex ratios predicted by the CTE model (light grey) and the DDC Model (dark grey) for each population (IL: Illinois, LA: Louisiana, SC: South Carolina). Points denote estimated sex ratios from an individual year.

**Figure 7:**
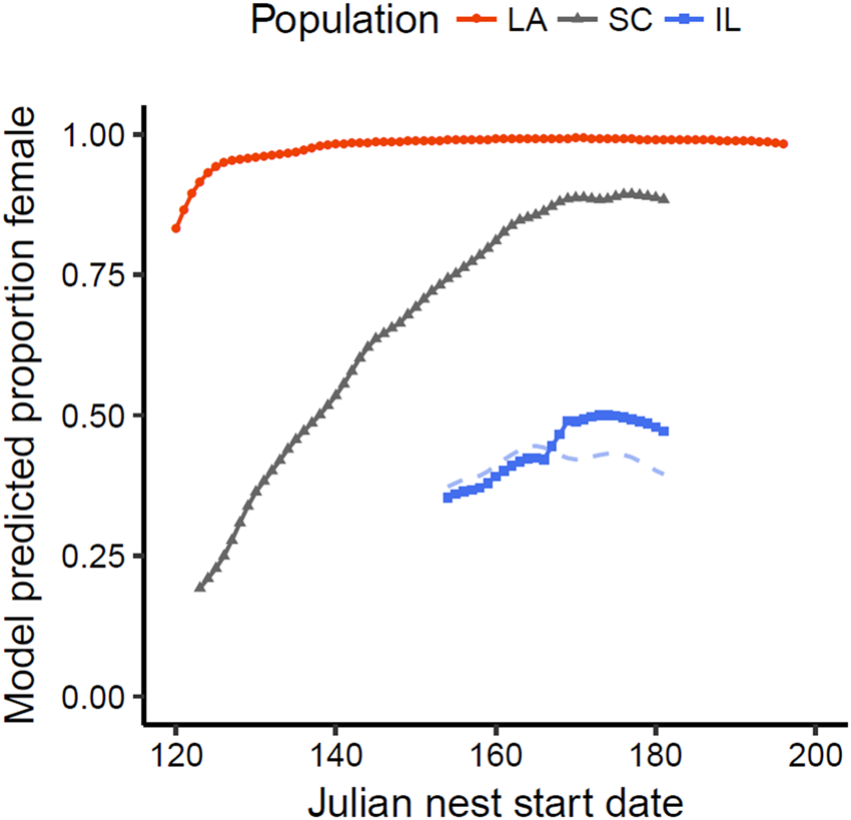
Sex ratios predicted for each population from the DDC Model across the nesting season. Sex ratios are averaged across all years by each nesting day, and a sliding 3-day average is depicted. Louisiana is shown with red circles, South Carolina with grey triangles, and Illinois with blue squares. Estimates for Louisiana and South Carolina use the population average sex ratio reaction norm (Fig. 3). Sex ratios in Illinois are portrayed using the population average sex ratio reaction norm (Fig. 3, dashed line), and the two season-specific reaction norms (Fig. 4, solid line with squares) to demonstrate the relative effect of the seasonal reaction norms on predicted sex ratios.

When examining overall population hatchling cohort sex ratios predicted by the DDC Model, Louisiana is predicted to be the most female biased, whereas South Carolina is only slightly female biased, and Illinois is slightly male biased (Fig. 6; reported as average proportion female; Louisiana: 0.92; South Carolina: 0.66; Illinois 0.44). The disparity between predictions made by the CTE model and the DDC Model seem to be more extreme in cooler conditions; specifically, the disparity is greatest in Illinois followed by South Carolina and Louisiana (Fig. 6). Populations also show considerable variation in their predicted sex ratios across their respective breeding seasons (Fig. 7). All populations demonstrate a seasonal increase in the production of females as ambient temperatures increase, with South Carolina increasing to the greatest extent.

Within the Illinois population, including the two season-specific sex ratio reaction norms to take maternal oestrogen deposition into account, seems to reinforce the seasonal increase in the production of females observed in other populations (Fig. 7).

## Discussion

By using more realistic thermal conditions (i.e., daily temperature fluctuations and simulated heat waves), we were able to demonstrate that sex ratios in a vertebrate with TSD can be modified by exposure to very short bouts of increased incubation temperatures. In some embryos, ovarian differentiation was triggered by a modest increase in incubation temperature of 2.5 °C for only five days. Surprisingly, early season eggs from all three populations exhibited a similar responsiveness despite different local climates, and in the only population in which it was examined, late season eggs were even more responsive. Using these new empirical findings, a model was developed to estimate sex ratios from field thermal traces that hones in on the number of days embryos are exposed to female-producing temperatures within the TSP. This model outperforms previously used methods that typically use temperatures from the entire TSP. This model was applied to thermal traces from each studied population to characterize predicted inter-population and seasonal differences across years.

### Empirical Study

We exposed embryos to varying durations of warm, sinusoidal thermal fluctuations to estimate the duration of exposure necessary to trigger ovary determination. Perhaps the most striking result from the empirical study is that only relatively short exposures to warm temperatures were necessary to trigger female development. Under the incubation conditions used in this study, *T. scripta* required 7.9 days of exposure to female producing conditions to achieve a 50:50 sex ratio, and after 5 days of exposure 16% females were produced, indicating that even brief exposures to warm temperatures can trigger ovary development in at least some individuals. Though embryonic sex may be labile throughout the entire TSP^19^, it is now clear that sex can be determined over just a few days within the TSP^40^. These more ecologically relevant conditions help clarify why sex ratios predicted from temperatures averaged across the entire incubation period or even only during the middle third of development may not accurately predict actual sex ratios^41^. That embryos are considerably more sensitive to female producing temperatures than originally thought, not only significantly alters our understanding of TSD, but should greatly improve sex ratio estimates under current and predicted climates. These findings more broadly underscore the importance of taking into account ecologically relevant thermal conditions in temperature sensitive responses.

Populations did not differ in the duration of exposure to female producing conditions necessary to trigger ovary development. The data do, however, help reconcile how females are produced under such cool conditions in the Illinois population, where average incubation temperatures do not rise above the Tpiv, and on average, there are only 8 days per year where maximum July temperatures rise above the Tpiv (1993–2016)^34^. Interestingly, in light of the current findings, this brief window of warm temperatures creates the potential for the production of at least some females in most years.

Late season embryos required less exposure to female producing conditions to produce a 50:50 sex ratio than early season embryos within the Illinois population (~6.5 versus 8.5 days). This is likely driven, at least in part, by maternal oestradiol deposition. In *T. scripta* and *C. picta*, there is a seasonal increase in maternally derived oestrogen concentrations in yolks, and this corresponds to the increased production of females late in the nesting season^35,42^. Research suggests that the physiological signal that triggers ovary development is oestradiol accumulation in the gonads^21,22^. Warmer incubation temperatures have been shown to decrease the methylation of the promoter genes that induce aromatase activity (which converts androgens to oestrogens) in the gonad^22^. By increasing aromatase production, oestradiol production increases^20^), and if enough oestradiol is produced to reach the signal accumulation threshold, ovary development results. We propose that late season clutches have a “head start” in accumulating oestradiol, and as a result, require less exposure to female producing temperatures to trigger ovary development. While there is still more work to do on elucidating the underlying mechanisms, studies that investigate oestradiol accumulation in the gonad (and other aspects of thermal signal accumulation described above) under incubation conditions that systematically vary exposure to warm conditions will further inform how sex determining signals respond to highly variable field conditions.

### Modeling Study

The DDC Model compares the number of days embryos experience CTEs that exceed the Tpiv to the reaction norm derived from the incubation study to calculate sex ratios. The DDC Model outperforms historically used methods of estimating sex ratios from field thermal traces. The biggest advance of the DDC Model is the ability restrict estimates to the days where female determination is likely to occur. Consider a scenario where only eight days out of a 30-day TSP are above the Tpiv. The findings from the empirical study herein suggest that female differentiation should be triggered in half of the hatchlings. The DDC Model predicts sex ratios based on those eight warm days, and would predict approximately 50% females. In contrast, historical methods like the average or the CTE predict sex from an aggregate statistic based on the temperatures from the entire TSP, which may include days outside of when sex has already been determined, ultimately underestimating the proportion of females produced. It is important to note that the CTE accurately estimates sex ratios under consistent sinusoidal fluctuations^29^ in the lab, and a degree-hour CTE approach^41^ accurately estimates sex ratios in the field when daily CTEs during the TSP do not cross the Tpiv (i.e. are entirely male- or female-producing conditions). However, these models begin to lose resolution under more variable thermal conditions experienced in the field, where temperatures during the TSP are both above and below the Tpiv^41^. Indeed, Georges *et al*.^41^ described that empirical evidence characterizing the amount of exposure to female-producing conditions necessary to trigger ovary development would be key to more accurately estimating sex ratios under variable field temperatures. These are precisely the data our incubation study provides, and are the foundation of the DDC Model. Integrating a more physiologically relevant understanding of how embryos respond to thermal fluctuations ultimately enhances the accuracy and ease of predicting sex ratios in the field.

Applying the DDC Model to temperature traces across years at each of our focal populations revealed a predicted latitudinal cline in average hatchling cohort sex ratios. These predictions mimic latitudinal patterns in the observed sex ratios of sea turtle populations^39^. Unfortunately, insufficient field data on hatchling cohort sex ratios in our species prohibits a direct comparison of the model predictions to natural observations. Should a latitudinal sex ratio pattern exist, results from the empirical study suggest that these patterns would be driven by differences in climate rather than physiological differences among populations.

Because the Louisiana population is predicted to be considerably female biased, the attention must now turn to the production of males in the south. Results from the DDC Model suggest that southern populations likely rely on cool early season nest temperatures and inter-annual variation in temperatures to produce males. Temperature-driven seasonal shifts in sex ratios have been observed in other TSD species^43^. Further, the influence of biased sex ratios may be alleviated if males mate multiply every breeding season and females take years off between breeding^44^, a breeding pattern that has been observed in sea turtles^44–47^ and *T. scripta* ^48^. The extensive lifespan of turtles, which results in males remaining in the breeding population over many years^47^, may further buffer populations from consecutive warm years that produce mostly to only females. From a conservation perspective, this suggests that in species of concern, nest protection efforts should focus on nests laid early in the season and in cooler-than-average years to protect the nests most likely to produce males, at least for populations in the south.

Integrating results from the empirical study into the DDC Model, demonstrates that the Illinois population exhibits a seasonal increase in the production of females underlain by both endogenous (likely maternal oestrogens) and exogenous (seasonal increase in temperature) factors. That maternal deposition of oestrogens and embryonic susceptibility to these oestrogens reinforces a seasonal shift in sex ratios driven by temperature, suggests a selection history leading to adaptive matches among offspring sex, incubation temperature (and therefore other temperature-dependent phenotypic traits), and season^35,43^. Further research is needed to determine if other populations experience a similar increase in maternal oestrogen concentrations in egg yolks across the season, and if embryos are similarly thermally responsive to those oestrogens.

There are several questions remaining that will be integral to refining the DDC Model. How much does the duration of exposure to female producing temperatures change with cooler ‘background’ temperatures? Does sensitivity to these ‘heat waves’ changes across the TSP? Do heat waves need to be consecutively experienced to have similar effects on ovary determination? Addressing these questions will improve the DDC Model and increase the ability to predict sex under more dynamic field temperatures.

## Conclusions

These findings highlight the importance of considering ecologically relevant thermal conditions in both empirical and theoretical research. A better characterization of how biological processes and organisms respond to realistic temperatures is central to the advancement of thermal biology and the improvement of thermal models. Such an endeavor will aid conservation efforts of thermally sensitive species who will continue to face challenges posed by climate change^49^.

## Methods

### Empirical Study

#### Egg Collection

For the population comparison, clutches of eggs were collected at the beginning of the respective nesting seasons from three different *T. scripta* populations in Louisiana, South Carolina, and Illinois. Twenty-nine clutches of eggs were collected on April 19, 2016 from Concordia Turtle Farm, LLC (Jonesville, LA), and 17 clutches of eggs were collected from the Department of Energy’s Savannah River Site near Aiken, South Carolina between May 3-4, 2016. In Illinois, 19 clutches of eggs were collected between May 31-June 1, 2016 at the Department of Natural Resources’ Banner Marsh State Fish and Wildlife Area (Canton, IL). All eggs were collected either by excavating freshly laid nests or by inducing gravid females with oxytocin in the lab^35^.

For the seasonal comparison within the Illinois population only, we collected 18 additional clutches laid late in the nesting season (June 14–16, 2016)^14–16^. Previous work demonstrates that late season clutches in this population have significantly higher concentrations of maternally derived yolk oestradiol and are more likely to produce female hatchlings than early season clutches^35^.

#### Incubation

Following collection, eggs from all populations were stored at room temperature in moist vermiculite before being transported to Illinois State University and incubated under common garden conditions. All eggs were placed into incubators within one to four days of being laid. Because sex is determined during the middle third of development^19^, it is unlikely that slight variation in pre-incubation storage time or temperatures affected hatchling sex ratios. Within an incubator, eggs were placed in small plastic containers with moist vermiculite, which were maintained at approximately −150 kPa. Eggs were randomly assigned to an incubation treatment in a split-clutch design to account for relatedness of individuals. Additionally, clutches and eggs within a treatment were split across all female producing (n = 3) (IPP 110 Plus and IPP 400, Memmert GmbH + Co.KG, Schwabach, Germany) and male producing (n = 2) (Fisher Isotemp Model 307 C, Fisher Scientific, Pittsburg, PA) temperature-programmed-incubators to account for incubator effects. Boxes were regularly rotated within incubators. Incubation period was measured as the amount of time from when eggs were put into the incubator until pipping (first eggshell breach).

The incubation regimes were designed to detect differences among populations and between seasons in the duration of exposure to female producing conditions necessary to produce 50:50 sex ratios (Table 1, Fig. 2). For the first twenty days, all eggs were incubated under a daily sinusoidal fluctuation of 27.0 ± 3 °C (“male producing condition”); incubation at 27.0 ± 3 °C for the entirety of development produces only male hatchlings^35^. On day 20 of incubation, in all groups except the control group, eggs were carefully and quickly moved to incubators with a daily sinusoidal fluctuation of 29.5 ± 3 °C (“female producing condition”). Incubation at 29.5 ± 3 °C for the entirety of development produces only female hatchlings^35^. Eggs from the control group were relocated within the incubator to control for egg movement, while all other eggs were placed in the female producing condition for 5, 9, 12, 15, or 20 days, after which they were returned to the male producing condition for the remainder of incubation. The Louisiana and South Carolina populations also had 25- and 30-day treatments. Upon observing initial sex ratios in the Louisiana population showing all females after a 20-day exposure, we were able to substitute the 25 and 30-day exposures for 7 and 10-day exposures in order to increase temporal resolution within the Illinois population. In the Illinois early season group only we also added 3-day and 6-day treatments to enhance resolution to better determine the minimum number of days required at the female producing condition for ovary development to occur. In each treatment, the number of eggs included varied slightly: Louisiana n = 19–22, South Carolina n = 13–14, and Illinois n = 19–22. We chose day 20 of incubation to begin exposure to the warm condition since this roughly corresponds to the beginning of the TSP^19^. Several embryos from each population were dissected on this date to confirm similar embryonic stages of development (stage 14–15 in all populations and both seasons)^15^. These manipulations produced a series of incubation treatments that varied in average incubation temperature and CTE across the entire developmental period (Table 1).

#### Hatchling Care and Sex Identification

Starting at about week seven of incubation, eggs were monitored daily for hatching. After hatching, all turtles were maintained in individual containers with water at room temperature, and containers were cleaned weekly. Hatchlings were reared for ~6 weeks to allow the gonads to fully differentiate to enable accurate sex identification. Hatchlings were euthanized, and sex was identified via macroscopic examination of the gonads by A. Wilson Carter^35^. All hatchling work was carried out in accordance to method approved by Illinois State University’s Institutional Animal Care and Use Committee (IACUC).

#### Yolk Oestradiol Quantification

Because eggs with elevated yolk oestradiol concentrations are more likely to produce female hatchlings in *T. scripta* ^35^, one egg from each clutch was frozen within 24 hours of oviposition to quantify yolk oestradiol concentrations via radioimmunoassay (RIA)^50,51^. Eggs were thawed and separated into shell, albumen, and yolk components. For the RIA, the entire yolk was homogenized and a 50 mg sample was taken, and diluted in 100 µl of distilled water. To each sample, 2000 cpm of tritiated oestradiol (PerkinElmer Life and Analytical Sciences, Boston, MA) was added and samples were then homogenized with glass beads and refrigerated overnight (4 °C). We also prepared four standard samples with 250 pg of oestradiol (Sigma-Aldrich, Inc., St. Louis, MO) and two blank samples of distilled water only. Our extraction solution was comprised of petroleum ether and diethyl ether in a 30:70 ratio. Steroids were extracted with two rounds of 3 mL of extraction solution, dried under nitrogen gas, and reconstituted in 90% ethanol. Samples were stored at −20 °C overnight. Samples were then centrifuged for 5 min at ~2000 rpm to pellet neutral lipids, decanted and dried under nitrogen gas. Samples were resuspended in 500 µl of 10% ethyl acetate in isooctane and fractionated in chromatography columns using the 20 and 40% fractions of ethyl acetate in isooctane. Previous pilot samples determined that using only the 20 and 40% fractions improved oestrogen recoveries. The 40% fraction was collected, dried under nitrogen gas, resuspended in 550 µl of phosphate buffer saline with gelatin (PBSG), and refrigerated overnight. Samples were then divided to measure recoveries (100 µl) and to make duplicates (200 µl each) to be run through a competitive binding RIA using an E_2_ specific antibody (Biogenesis Inc., Kingston, NH). Oestradiol concentrations for each sample were calculated using a standard curve that ranged from 1.95–500 pg. The cpm were averaged across duplicates and corrected for individual sample recovery and initial yolk sample mass (average recovery 44%).

#### Statistics

Oestradiol Concentrations. To test for population or seasonal differences in yolk oestradiol, we used two separate mixed model ANCOVAs in SAS (v 9.4), with population or season as the main effect, assay as a random effect, and sample recovery as a covariate.

#### Population or Seasonal Differences in Sex Ratio Reaction Norms

The effect of exposure duration to female producing conditions on the proportion of females produced was estimated using a generalized log-logistic model in R 3.3.3^52^ with the *drc* package^53^. The best fitting model had the following two-parameter log-logistic function:

1 $$f(x)=\frac{1}{(1+\exp (a+\,\mathrm{log}(x)-\,\mathrm{log}(b))))}$$

This dose-response model is parameterized using a unified structure with a coefficient *a* denoting the steepness of the model (i.e. the Hill coefficient) and *b* denoting the effective dose producing 50% females (i.e. ED 50)^53^. The model also relies on parameterizing the upper and lower asymptotes of the response, which are constrained by 0 and 1 in this system, thus rendering the model to two parameters. A three-parameter version of the model was also fitted that, in addition to the parameters described above, also included asymmetry of the function around the point of inflection. The models did not significantly differ (Likelihood ratio test: χ^2^_1_ = 0.92, p = 0.34), and the two-parameter model had lower Akaike’s Information Criterion (AIC) values (297.5 versus 298.6) and narrower standard errors (Two-parameter model: *a:* 0.39, *b:* 0.30; Three-parameter model: *a:* 1.68, *b:* 2.12). We therefore proceeded with the more parsimonious, two-parameter model (Eq. 1). To test for population differences in the sex ratio reaction norms, we allowed parameters (*a, b*) to vary by population (Louisiana, South Carolina, Illinois). Similarly, to test for season differences within Illinois the parameters were estimated independently for each season (early or late). We compared these models to the model that only specified incubation duration treatment (above) with likelihood ratio tests and AIC. This method allowed us to compare the entire reaction norm of each population or season derived from all treatments, including treatments that were not represented across all groups.

### Modeling Study

#### Summary of Modeling Approach

We wanted to determine if basing a model on our empirical data that explicitly characterizes the response of a biological process to thermal variability could improve the natural applicability of sex ratio predictions. We developed a model, the Daily Duration with CTE (DDC) Model, that estimates sex based on the duration of exposure to female-producing conditions. We (I) examined if our approach better estimates sex ratios in the field compared to historical approaches (average and CTE), (II) characterized predicted sex ratios from each of our populations across time, and (III) compared the relative contribution of season-specific maternal oestrogen deposition (in the form of season-specific sex ratio reaction norms) to population sex ratios under field temperatures.

#### Estimating Sex Ratios Using the CTE

We calculated CTEs and estimated the resulting sex ratios as a metric of comparison for the DDC Model. The DDC Model also relies on the CTE equations to identify the days within the TSP that embryos are exposed to female-producing conditions. Similar equations were used in both the CTE and DDC Model. Using daily maximum, minimum, and average temperatures from soil temperature trace data, we calculated CTEs^29^ as:

2 $$CTE=R\cdot \,\cos [t^{\prime} ]+M$$

3 $$\,t^{\prime} =\frac{\pi }{2}-\frac{R}{M-T0}\,\sin \,[t^{\prime} ]$$

M is the average daily temperature and *2 R* is the daily thermal range (and therefore *R* is the amplitude of the daily thermal fluctuation). *T0* is the developmental zero constant, the species-specific minimal temperature where embryonic development ceases; in *T. scripta* this value is 16 °C^31^. Lastly, *t*′ is the intermediate value eliminated from the equations when solving for CTE and is expressed in radians.

The proportion of females predicted to be produced based on the average CTE during the TSP was calculated using a piece-wise function derived from previously published data^35^. A CTE greater than or equal to 29.8 °C was assigned a value of 1 (100% female production), and a CTE less than or equal to 28 °C was assigned a value of 0 (0% female production)^35^. CTEs between these values were calculated based on the following function and parameter estimates found to be the best fit for the previous data:

4 $$f(x)=c+\,\frac{d-c}{{(1+\exp (a\ast (\mathrm{log}(x)-\mathrm{log}(b)))}^{g}}$$

where *a* and *b* are as Eq. 1 above, *c* is the lower asymptote, *d* is the upper asymptote, and *g* estimates asymmetry around the inflection point, with parameter estimates of: *a:* −9.48*, b:* 27.58*, c:* −0.01, *d*: 1.05*, g:* 13.18.

#### The DDC Model

CTEs were calculated for each day and assigned a score of 1 if it was greater than the species Tpiv (28.5 °C; 35) and a score of 0 if it was below. We used these scores to sum the number of days temperatures rose above the Tpiv during the estimated TSP (days 20–50). Eq. 1 was then implemented to incorporate these values and estimates from the fitted model of the empirical data to estimate the proportion of females produced. This process was repeated for nest start dates spanning the population-specific nesting season: Illinois: June 1- June 30 (*pers. obs*.), South Carolina: May 1–June 30^48^, Louisiana: April 15–July 15^48^. For example, for a nest laid on June 1, the number of days the daily CTE was above the Tpiv was summed between June 21 and July 21, and this count was included in Eq. 1 to estimate the proportion of female offspring. The annual proportion of females produced in the hatchling cohort was estimated by averaging the daily estimates from the entire nesting season each year. Though nesting frequency is likely not uniformly distributed across the nesting season, insufficient data describing the temporal nesting patterns in each population prohibits any such parameter from being included in the current model.

#### Determining the Predictive Ability of the DDC Model

To validate the use of our model, we tested our methods on a long-term study population of *C. picta*, where hatchling cohort sex ratios are estimated annually by macroscopic examination of the gonads in a subset of nests^23–25^. This population is located along the Illinois River in north-western Illinois and has been the focus of study by F. J. Janzen (Iowa State University) for over 20 years. Observed sex ratios from 1990–2013 were estimated from Fig. 1. of Refsnider *et al*.^25^ with a standardized method in ImageJ (V 1.48, NIH USA). Briefly, the figure was uploaded into ImageJ, and the height (in pixels) of each data point was measured from the x-axis (n = 3 times each, then averaged). The average height of the data point was converted from pixels to a percent male by determining the number of pixels in a 10% increase on the y-axis. This method was validated by comparing the estimated percent male using ImageJ to a subset of the sex ratio in data reported in Schwanz *et al*.^24^. We used soil temperature trace data from a nearby site (ISWS, Freeport 1990–2016)^34^. We compared observed sex ratios (on the y-axis) to the sex ratios predicted from the CTE and the DDC Model (on the x-axis)^54^. We compared these models and their explanatory power using a Cox Test, J Test, and Encompassing Test. All three tests returned highly coincident results, so for simplicity, we only report results from the Encompassing Test.

#### Estimating Sex Ratios

We applied the DDC Model to soil temperature trace data sets from each of our three study populations: Illinois (Peoria 1993–2016)^34^, South Carolina (data obtained from the DOE’s Savanna River National Lab, Aiken 1990–2016), and Louisiana (Hammond 2002–2014)^55^. Illinois and Louisiana temperatures are taken at a depth of 10.1 cm and South Carolina temperature readings are at a depth of 15.25 cm. Because populations did not significantly differ in parameter estimates (see results), we utilized a single reaction norm derived from all populations to estimate the proportion of females produced. In limited cases where daily temperatures were missing during the TSP of a particular nest start date, the CTE was averaged across available days and the overall number of days above the CTE was above the Tpiv was extrapolated proportionally from the available data.

Within the Illinois population, sex ratio reaction norms significantly differed between seasons (see results), in a manner concordant to maternal oestrogen deposition. As such, we estimated sex ratios within the Illinois population using two season-specific sex ratio reaction norms. In the model, nests laid between June 1- June 15 utilized the early season reaction norm, and nests laid between June 16-June 30 utilized the late season reaction norm (see results for parameter estimates).

### Data Availability

The datasets generated an analysed during the current study are available from the corresponding author on reasonable request.
